# Supplementary material for: Why aphid virus retention needs more attention: Modelling aphid behaviour and virus manipulation in non-persistent plant virus transmission
Source: PLoS Comput Biol. 2024 Oct 1;20(10):e1012479. doi: 10.1371/journal.pcbi.1012479 (PMC11469505; doi:10.1371/journal.pcbi.1012479)
Supplement: S1 Appendix — This appendix shows that the MIP-BAR model reduces to the BAR model when the aphid dynamics are held at pseudo-steady state, when ρ = 1 (i.e. without Multiple Infective Probes). (PDF) [file pcbi.1012479.s001.pdf]

Appendix S1: Relating the MIP-BAR and BAR models

We show here that the MIP-BAR model reduces to the BAR model when the aphid dynamics are held at pseudo-steady state, at least when $\rho = 1$ (i.e., without Multiple Infective Probes).

1 Models

1.1 BAR model

The BAR model (Equations 11-12 in main text) is

$$\frac{dI}{dt} = \theta A x(\tilde{i}) - \Gamma I, \tag{S1}$$

where

$$x(\tilde{i}) = \frac{ab\tilde{i}(1-\varepsilon\omega)(1-\tilde{i})}{(1-\tilde{i})\omega + \tilde{i}\varepsilon\omega}, \tag{S2}$$

and where

$$\tilde{i} = \frac{vI}{H-I+vI} \text{ and } 1-\tilde{i} = \frac{H-I}{H-I+vI}. \tag{S3}$$

The model can be written as

$$\frac{dI}{dt} = \theta \frac{Aab(1-\varepsilon\omega)vI(H-I)}{\omega(H-I+vI)(H-I+v\varepsilon I)} - \Gamma I. \tag{S4}$$

1.2 MIP-BAR model

The MIP-BAR model (Equations 24-25 in main text) is

$$\frac{dI}{dt} = \frac{(H-I)bZ}{\omega\eta(H-I+v\varepsilon I)} - \Gamma I, \tag{S5}$$

$$\frac{dZ}{dt} = \frac{vI\alpha(1-\varepsilon\omega)(A-Z)}{\omega\eta(H-I+v\varepsilon I)} - \frac{(H-I)[\rho+(1-\rho)\omega] + vI[\rho(1-\alpha(1-\varepsilon\omega)) + (1-\rho)\varepsilon\omega]}{\omega\eta(H-I+v\varepsilon I)} Z. \tag{S6}$$

With $\rho = 1$ the model simplifies to:

$$\frac{dI}{dt} = \frac{(H-I)bZ}{\omega\eta(H-I+v\varepsilon I)} - \Gamma I, \tag{S7}$$

$$\frac{dZ}{dt} = \frac{vI\alpha(1-\varepsilon\omega)(A-Z)}{\omega\eta(H-I+v\varepsilon I)} - \frac{H-I+vI(1-\alpha(1-\varepsilon\omega))}{\omega\eta(H-I+v\varepsilon I)} Z. \tag{S8}$$

2 The MIP-BAR model (when $\rho = 1$) at aphid pseudo-equilibrium reduces to the BAR model

Consider a restriction of the MIP-BAR model such that the density of aphids, Z, is restricted to lie on the relevant null cline, i.e., $dZ/dt = 0$. This corresponds to assuming the dynamics of the number of infective aphids are much faster than the epidemiological dynamics affecting the number of infected plants.

In this case, since

$$0 = \frac{\nu I\alpha(1-\varepsilon\omega)(A-Z) - [H - I + \nu I(1-\alpha(1-\varepsilon\omega))]Z}{\omega\eta(H - I + \nu\varepsilon I)}, \tag{S9}$$

the number of infective aphids can be written in terms of the number of infected plants, with

$$Z = \frac{\nu I\alpha(1-\varepsilon\omega)A}{H - I + \nu I}. \tag{S10}$$

Inserting this expression for Z as a function of I into Equation S7 leads to

$$\frac{dI}{dt} = \frac{(H-I)b}{\omega\eta(H - I + \nu\varepsilon I)}\frac{\nu I\alpha(1-\varepsilon\omega)A}{H - I + \nu I} - \Gamma I, \tag{S11}$$

$$= \frac{1}{\eta}\frac{A\alpha b(1-\varepsilon\omega)\nu I(H-I)}{\omega(H - I + \nu I)(H - I + \nu\varepsilon I)} - \Gamma I. \tag{S12}$$

But since θ is the rate of initiating a feeding dispersal in the BAR model, and η is the average length of an aphid feed in the MIP-BAR model, the two parameters are related via $1/\eta = \theta$, and so – comparing Equation S4 and Equation S12 – the two models are clearly equivalent.

Note that, irrespective of the relative speed of aphid *vs.* plant dynamics, this means that when $\rho = 1$, the disease (I) equilibria of the two models must be identical. However, transient aphid dynamics in the MIP-BAR model mean that epidemic trajectories of the two models will not (necessarily) match, although if aphid dynamics are relatively rapid the concordance between the full trajectories from both models is likely to be relatively high.
