# Supplementary material for: Why aphid virus retention needs more attention: Modelling aphid behaviour and virus manipulation in non-persistent plant virus transmission
Source: PLoS Comput Biol. 2024 Oct 1;20(10):e1012479. doi: 10.1371/journal.pcbi.1012479 (PMC11469505; doi:10.1371/journal.pcbi.1012479)
Supplement: S2 Appendix — (PDF) [file pcbi.1012479.s002.pdf]

Appendix S2: Basic reproduction numbers for the three models

1 MIP-BAR model

Both compartments of the MIP-BAR model (2 compartment, rather than 4 compartment version) involve infection (note these are Equations 24-25 in the main text)

$$\frac{dI}{dt} = \frac{(H-I)bZ}{\omega\eta(H-I+v\varepsilon I)} - \Gamma I, \tag{S1}$$

$$\frac{dZ}{dt} = \frac{vIa(1-\varepsilon\omega)(A-Z)}{\omega\eta(H-I+v\varepsilon I)} - \frac{(H-I)[\rho+(1-\rho)\omega]+vI[\rho(1-a(1-\varepsilon\omega))+(1-\rho)\varepsilon\omega]}{\omega\eta(H-I+v\varepsilon I)}Z. \tag{S2}$$

Following the standard method for Next Generation Matrix calculations [1], we define F as the matrix of infection terms (i.e., entering the state) and V as the matrix of loss-of-infection terms (i.e., leaving the state).

$$F = \begin{bmatrix} \dfrac{(H-I)bZ}{\omega\eta(H-I+v\varepsilon I)} \\ \dfrac{vIa(1-\varepsilon\omega)(A-Z)}{\omega\eta(H-I+v\varepsilon I)} \end{bmatrix}, \tag{S3}$$

$$V = \begin{bmatrix} \Gamma I \\ \dfrac{(H-I)[\rho+(1-\rho)\omega]+vI[\rho(1-a(1-\varepsilon\omega))+(1-\rho)\varepsilon\omega]}{\omega\eta(H-I+v\varepsilon I)}Z \end{bmatrix}. \tag{S4}$$

The Jacobians of these matrices are

$$J_F = \begin{bmatrix} \dfrac{-bZv\varepsilon H}{\omega\eta(H-I+v\varepsilon I)^2} & \dfrac{(H-I)b}{\omega\eta(H-I+v\varepsilon I)} \\ \dfrac{va(1-\varepsilon\omega)(A-Z)H}{\omega\eta(H-I+v\varepsilon I)^2} & \dfrac{-vIa(1-\varepsilon\omega)}{\omega\eta(H-I+v\varepsilon I)} \end{bmatrix}, \tag{S5}$$

$$J_V = \begin{bmatrix} \Gamma & 0 \\ \dfrac{ZHv[\rho(1-a(1-\varepsilon\omega))-\varepsilon\rho]}{\omega\eta(H-I+v\varepsilon I)^2} & \dfrac{(H-I)[\rho+(1-\rho)\omega]+vI[\rho(1-a(1-\varepsilon\omega))+(1-\rho)\varepsilon\omega]}{\omega\eta(H-I+v\varepsilon I)} \end{bmatrix}, \tag{S6}$$

and when evaluated at the disease-free equilibrium, $I=0$, $Z=0$, they become

$$J_F = \begin{bmatrix} 0 & \dfrac{b}{\omega\eta} \\ \dfrac{va(1-\varepsilon\omega)A}{\omega\eta H} & 0 \end{bmatrix} \text{ and } J_V = \begin{bmatrix} \Gamma & 0 \\ 0 & \dfrac{\rho+(1-\rho)\omega]}{\omega\eta} \end{bmatrix}. \tag{S7}$$

The basic reproduction number, R_0, is the spectral radius (i.e., value of the leading eigenvalue) of the Next Generation Matrix

$$M = J_F J_V{}^{-1} = \begin{bmatrix} 0 & \dfrac{b}{\rho + (1-\rho)\omega} \\ \dfrac{v a (1-\epsilon\omega) A}{\omega \eta H \Gamma} & 0 \end{bmatrix}, \tag{S8}$$

and so

$$R_0^2 = \frac{b v a (1-\epsilon\omega) A}{\omega \eta H \Gamma [\rho + (1-\rho)\omega]}. \tag{S9}$$

This matches the expression given in the main text (Equation 28).

2 MIP model

Again, both compartments of the MIP model (2 compartment, rather than 4 compartment version; Equations 5-6 in main text) involve infection, with

$$\frac{dI}{dt} = \phi b Z \frac{H-I}{H-I+vI} - \Gamma I, \tag{S10}$$

$$\frac{dZ}{dt} = \phi a (1-\varepsilon\omega)(A-Z) \frac{vI}{H-I+vI} - \tau Z. \tag{S11}$$

Repeating the calculation done above

$$F = \begin{bmatrix} \dfrac{\phi b Z (H-I)}{H-I+vI} \\ \dfrac{\phi a (1-\varepsilon\omega)(A-Z) vI}{H-I+vI} \end{bmatrix}, \tag{S12}$$

$$V = \begin{bmatrix} \Gamma I \\ \tau Z \end{bmatrix}, \tag{S13}$$

the Jacobians are

$$J_F = \begin{bmatrix} \dfrac{-\phi b Z H v}{(H-I+vI)^2} & \dfrac{\phi b (H-I)}{H-I+vI} \\ \dfrac{\phi a (1-\varepsilon\omega)(A-Z) v H}{(H-I+vI)^2} & \dfrac{-\phi a (1-\varepsilon\omega) vI}{H-I+vI} \end{bmatrix}, \tag{S14}$$

$$J_V = \begin{bmatrix} \Gamma & 0 \\ 0 & \tau \end{bmatrix}, \tag{S15}$$

and when evaluated at the disease-free equilibrium, $I = 0$ and $Z = 0$ these matrices become

$$J_F = \begin{bmatrix} 0 & \phi b \\ \dfrac{\phi a (1-\varepsilon\omega) A v}{H} & 0 \end{bmatrix} \text{ and } J_V = \begin{bmatrix} \Gamma & 0 \\ 0 & \tau \end{bmatrix}. \tag{S16}$$

Since the Next Generation Matrix is

$$M = \begin{bmatrix} 0 & \dfrac{\phi b}{\tau} \\ \dfrac{\phi a(1-\varepsilon\omega)Av}{H\Gamma} & 0 \end{bmatrix}, \tag{S17}$$

the basic reproduction number is

$$R_0^2 = \frac{\phi^2 ab(1-\varepsilon\omega)Av}{\tau H\Gamma}. \tag{S18}$$

Again this matches the expression given in the main text (Equation 27).

3 BAR model

The BAR model is one-dimensional, and so the mathematical machinery of the Next Generation Method is not required. As seen in Equations 11-12 in the main text, the model is

$$\frac{dI}{dt} = \theta A x(\tilde{i}) - \Gamma I, \tag{S19}$$

where

$$x(\tilde{i}) = \frac{ab\tilde{i}(1-\varepsilon\omega)(1-\tilde{i})}{(1-\tilde{i})\omega + \tilde{i}\varepsilon\omega}, \tag{S20}$$

and where

$$\tilde{i} = \frac{vI}{H-I+vI} \text{ and } 1-\tilde{i} = \frac{H-I}{H-I+vI}. \tag{S21}$$

The model can be written as

$$\frac{dI}{dt} = \frac{\theta Aab(1-\varepsilon\omega)vI(H-I)}{\omega(H-I+vI)(H-I+v\varepsilon I)} - \Gamma I, \tag{S22}$$

$$= \Gamma I\left(\frac{\theta Aab(1-\varepsilon\omega)v(H-I)}{\omega(H-I+vI)(H-I+v\varepsilon I)\Gamma} - 1\right), \tag{S23}$$

and so in the vicinity of the disease-free equilibrium ($I = 0$) the model reduces to

$$\frac{dI}{dt} \approx \Gamma\left(\frac{\theta Aab(1-\varepsilon\omega)v}{\omega H\Gamma} - 1\right)I, \tag{S24}$$

i.e., exponential growth of the number of infected plants at rate

$$\Gamma\left(\frac{\theta Aab(1-\varepsilon\omega)v}{\omega H\Gamma} - 1\right). \tag{S25}$$

This means

$$R_0 = \frac{\theta Aab(1-\varepsilon\omega)v}{\omega H\Gamma}, \tag{S26}$$

as given in the main text (Equation 26).

References

1. van den Driessche P. Reproduction Numbers of Infectious Disease Models. Infectious Disease Modelling. 2017;2(3):288–303. doi:10.1016/j.idm.2017.06.002.
