# Supplementary material for: Why aphid virus retention needs more attention: Modelling aphid behaviour and virus manipulation in non-persistent plant virus transmission
Source: PLoS Comput Biol. 2024 Oct 1;20(10):e1012479. doi: 10.1371/journal.pcbi.1012479 (PMC11469505; doi:10.1371/journal.pcbi.1012479)
Supplement: S3 Appendix — (PDF) [file pcbi.1012479.s003.pdf]

Appendix S3: With $\rho = 1$, the infectivity loss rate, $\tilde{\tau}$, in the MIP-BAR model becomes equal to the aphid dispersal rate, $\tilde{\phi}$, when $I = 0$

In the MIP-BAR model, the aphid dispersal rate, $\tilde{\phi}$, and aphid infectivity loss rate, $\tilde{\tau}$, are defined by (Equations 14 and 20 in the main text, respectively; see Table 2 in main text for parameter definitions)

$$\tilde{\phi} = \frac{S + vI}{\omega\eta(S + v\varepsilon I)}, \tag{S1}$$

$$\tilde{\tau} = \frac{S[\rho + (1-\rho)\omega] + vI[\rho(1 - a(1-\varepsilon\omega)) + (1-\rho)\varepsilon\omega]}{\omega\eta(S + v\varepsilon I)}. \tag{S2}$$

When $\rho = 1$, i.e. when there is no Multiple Infective Probes functionality, the expression for $\tilde{\tau}$ simplifies to

$$\tilde{\tau} = \frac{S + vI[1 - a(1-\varepsilon\omega)]}{\omega\eta(S + v\varepsilon I)}. \tag{S3}$$

When the epidemic dies out, $I = 0$ and $S = H$. In the case $\rho = 1$, these expressions simplify to

$$\tilde{\phi} = \frac{H}{\omega\eta H} = \frac{1}{\omega\eta}, \tag{S4}$$

$$\tilde{\tau} = \frac{H}{\omega\eta H} = \frac{1}{\omega\eta}. \tag{S5}$$

Therefore in this case, at $I = 0$, the aphid infectivity loss rate becomes equal to the dispersal rate. This makes intuitive sense, as with no I plants, and in the case $\rho = 1$ where there is a 100% chance of loss of infectivity when probing a S plant, any time an infective aphid disperses it is guaranteed to lose its infectivity. This is also shown in Figure S1, where, for all parameters, when disease incidence (grey line) is 0, the aphid infectivity loss (red line) and dispersal rate (blue) are indistinguishable, and equal to $1/\omega\eta$. With the exception of Figure S1a, where ω is being varied, $1/\omega\eta \approx 6$ under the default parameterisation (see Table 2, main text). Note the panels of Figure S1 are identical to their counterparts in the main text (but with only the MIP-BAR model rates, i.e., the green lines):

- Figure S1a = Figure 5a,b,c combined
- Figure S1b = Figure 5d,e,f combined
- Figure S1c = Figure 5g,h,i combined
- Figure S1d = Figure 6a,b,c combined

- Figure S1e = Figure 6d,e,f combined

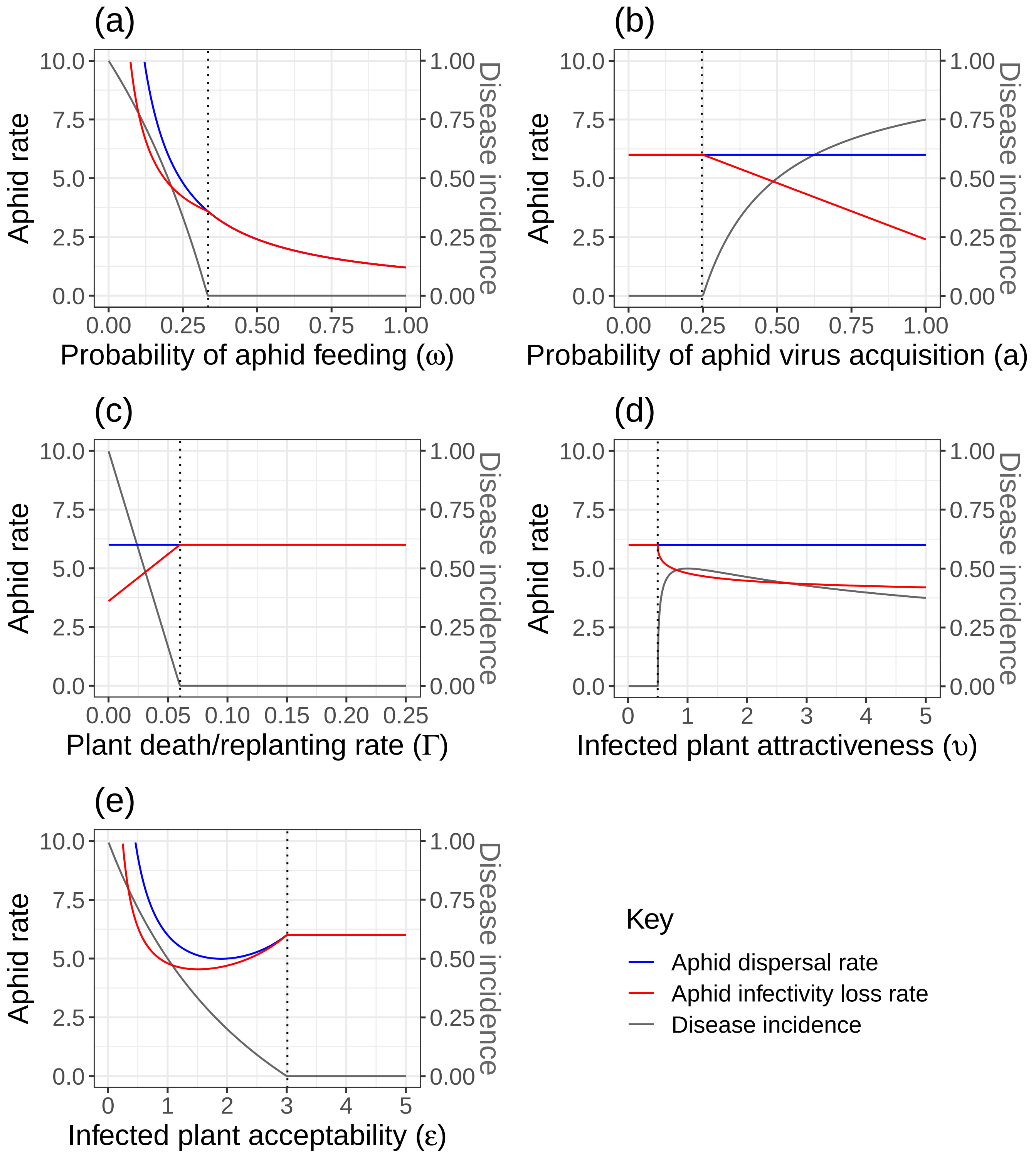

Figure S1: **Aphid infectivity loss rate ($\tilde{\tau}$) becomes equal to aphid dispersal rate ($\tilde{\phi}$) when disease incidence is 0 in the MIP-BAR model (when $\rho = 1$).** $\tilde{\phi}$, $\tilde{\tau}$, and disease incidence across values of model parameters: *(a)* Probability of aphid feeding, ω, *(b)* Probability of aphid acquiring virus from probing infected plant (a) *(c)* Plant death/replanting rate, Γ, *(d)* Infected plant attractiveness (v), a virus manipulation parameter, *(e)* Infected plant acceptability, a virus manipulation parameter. Disease incidence is the equilibrium proportion of infected plants (I/H). Vertical dotted black line on each panel shows the point at which disease incidence becomes 0 (equilibrium $I/H = 0$).
