# Supplementary material for: Why aphid virus retention needs more attention: Modelling aphid behaviour and virus manipulation in non-persistent plant virus transmission
Source: PLoS Comput Biol. 2024 Oct 1;20(10):e1012479. doi: 10.1371/journal.pcbi.1012479 (PMC11469505; doi:10.1371/journal.pcbi.1012479)
Supplement: S4 Appendix — (PDF) [file pcbi.1012479.s004.pdf]

Appendix S4: Adding Multiple Infective Probes (MIP) to the BAR model is analytically intractable

The model by Donnelly et al. [1] (the BAR model) assumes that aphids will always lose infectivity after their first probe of a healthy plant (although infectivity can be regained in a single round of dispersal if an infected plant is subsequently probed). Multiple infective probes (MIP) in the sense described in the main text of the current paper are therefore not possible.

Here we show that relaxing this assumption, and allowing infectivity to be retained by a viruliferous aphid which probes a susceptible plant, significantly increases the complexity of the Donnelly et al. [1] model. Indeed, adding this functionality makes it impossible to specify the model in closed form (although the relevant quantities to simulate the model can still be found numerically).

1 Including Multiple Infective Probes in the BAR model

The expression $x(\tilde{i})$ in the Donnelly et al. [1] model represents the mean number of transmissions per aphid feeding dispersal. This quantity enters the differential equation for the rate of change of the number of infected plants (Equation 11 in the main text), driving disease dynamics.

In Donnelly et al. [1], a closed form expression for $x(\tilde{i})$ is derived by finding a probability mass function (p.m.f.) for the number of transmissions per dispersal. Relaxing the assumption of one transmission per infective period necessitates additional states in the Markov chain which tracks aphid dispersal (Figure S1). Using the notation of Donnelly et al. [1], there are now eight rather than four probabilities with respect to feeding versus inoculating:

- $P_F^{S,X}$ = probability of the aphid feeding before inoculating (F), starting un-infective (X) on a healthy (S) plant
- $P_F^{S,Z}$ = probability of the aphid feeding before inoculating (F), starting infective (Z) on a healthy (S) plant
- $P_k^{S,X}$ = probability of the aphid inoculating before feeding (k), starting un-infective (X) on a healthy (S) plant
- $P_k^{S,Z}$ = probability of the aphid inoculating before feeding (k), starting infective (Z) on a healthy (S) plant
- $P_F^{I,X}$, $P_F^{I,Z}$, $P_k^{I,X}$ and $P_k^{I,Z}$ = the same probabilities as above but starting from an infected (I) plant

From these probabilities, the p.m.f. of the number of inoculations, n, per feeding dispersal can be calculated, as in Donnelly et al. [1]. If the probability of n inoculations during a feeding dispersal is P_n, components of the p.m.f. are linked by the following recursion

$$P_0 = \tilde{i}P_F^{I,X} + \tilde{s}P_f^{S,X}, \tag{S1}$$

$$P_n = (\tilde{i}P_k^{I,X} + \tilde{s}P_k^{S,X})(\rho P_k^{S,X} + (1-\rho)P_k^{S,Z})^{n-1}(\rho P_F^{S,X} + (1-\rho)P_F^{S,Z}). \tag{S2}$$

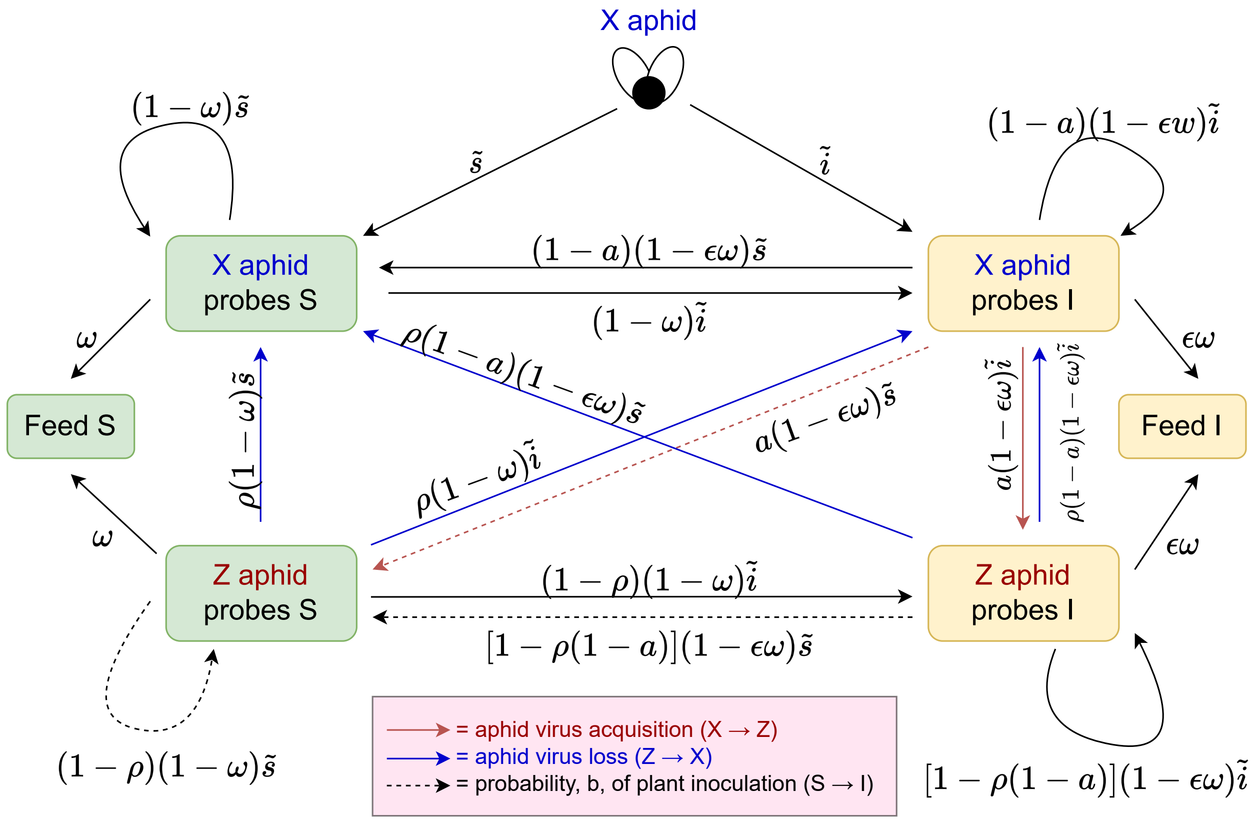

Figure S1: **Schematic showing the aphid feeding dispersal Markov chain if the assumption of only one infective probe is relaxed.** X = uninfective aphid, Z = infective aphid, S = susceptible plant, I = infected plant. Note $\tilde{i}$ and $\tilde{s}$ are the weighted probabilities of landing on an infected and healthy plant respectively. $\tilde{i} = vI/(S+vI)$ and $\tilde{s} = S/(S+vI) = 1-\tilde{i}$. ρ = probability of infectivity loss from probing. a = probability of virus acquisition from probing an infected plant. ω = probability of feeding on a plant. v = degree of virus-induced plant attractiveness. ε = degree of virus-induced plant acceptability.

Note that P_0 is the probability of no inoculations occurring. As all aphids enter a feeding dispersal having just fed, they are always un-infective (X), and so P_0 is the probability of an X aphid landing on an infected plant ($\tilde{i}$) then feeding before inoculating ($P_F^{I,X}$), or landing on a healthy plant and doing the same ($\tilde{s}P_F^{S,X}$). The probability of n inoculations is the probability an (X) aphid, when it starts a feeding dispersal, lands on an infected or healthy plant and then inoculates before feeding ($\tilde{i}P_k^{I,X} + \tilde{s}P_k^{S,X}$). It then leaves the S (just inoculated so soon to be I) plant, either still infective (with probability $1-\rho$) or uninfective, having lost its infectivity (with proability ρ),and goes through the same process of inoculating before feeding a further $n-1$ times, $(\rho P_k^{S,X} + (1-\rho)P_k^{S,Z})^{n-1}$, before finally feeding before inoculating ($P_F^{S,X} + P_F^{S,Z}$), ending the dispersal.

2 Analysis of the extended BAR model

The probability expressions that comprise the p.m.f. can be calculated by a Markov chain conditioning argument, as shown in Figure S1, and are given in Equations S3-S10. For example, for $P_F^{I,Z}$ (Equation S10), the expression is the probability an aphid feeds before inoculating, given that it is infective (Z) and has just landed and probed an infected (I) plant. At this point, the aphid could feed with probability $\epsilon\omega$, in which case feeding occurs before inoculation. If instead the aphid rejects the plant with probability $1-\epsilon\omega$, it will leave for a different plant. The arrows

in Figure S1 detail the probability of moving between two compartments. Which compartment it moves to (from the 'Z aphid probes I' compartment) depends on both whether it retained or lost the virus during probing, and whether it next lands on an S or I plant. For example, if the aphid lost the virus during probing (probability $\rho(1-a)$) and landed on another I plant (probability $\tilde{i}$), the probability of it now feeding before inoculating is now $P_F^{I,X}$, i.e. the probability of feeding before inoculating given an un-infective aphid on an infected plant. This is true for all possible transitions except for the case where the aphid doesn't lose infectivity, lands on an S plant, and inoculates the plant (probability $(1-\epsilon\omega)[1-\rho(1-a)]\tilde{s}b$). As this represents an inoculation event, the aphid therefore does not feed before inoculation, and so is multiplied by 0 in Equation S10.

Applying similar logic for all transitions leads to the following set of four equations which specify the probabilities in the case where the aphid is on an S plant (having just probed):

$$P_k^{S,X} = \omega \cdot 0 + (1-\omega)\tilde{s}P_k^{S,X} + (1-\omega)\tilde{i}P_k^{I,X}, \tag{S3}$$

$$P_F^{S,X} = \omega \cdot 1 + (1-\omega)\tilde{s}P_F^{S,X} + (1-\omega)\tilde{i}P_F^{I,X}, \tag{S4}$$

$$\begin{aligned} P_k^{S,Z} = &\omega \cdot 0 + \rho(1-\omega)\tilde{s}P_k^{S,X} + \rho(1-\omega)\tilde{i}P_k^{I,X} + (1-\rho)(1-\omega)\tilde{s}b \cdot 1 + \\ &(1-\rho)(1-\omega)\tilde{s}(1-b)P_k^{S,Z} + (1-\rho)(1-\omega)\tilde{i}P_k^{I,Z}, \end{aligned} \tag{S5}$$

$$\begin{aligned} P_F^{S,Z} = &\omega \cdot 1 + \rho(1-\omega)\tilde{s}P_F^{S,X} + \rho(1-\omega)\tilde{i}P_F^{I,X} + (1-\rho)(1-\omega)\tilde{s}b \cdot 0 + \\ &(1-\rho)(1-\omega)\tilde{s}(1-b)P_F^{S,Z} + (1-\rho)(1-\omega)\tilde{i}P_F^{I,Z}. \end{aligned} \tag{S6}$$

The following equations correspond to an aphid which is on an I plant (having just probed):

$$\begin{aligned} P_k^{I,X} = &\epsilon\omega \cdot 0 + (1-a)(1-\epsilon\omega)\tilde{s}P_k^{S,X} + (1-a)(1-\epsilon\omega)\tilde{i}P_k^{I,X} + a(1-\epsilon\omega)\tilde{s}b \cdot 1 + \\ &a(1-\epsilon\omega)\tilde{s}(1-b)P_k^{S,Z} + a(1-\epsilon\omega)\tilde{i}P_k^{I,Z}, \end{aligned} \tag{S7}$$

$$\begin{aligned} P_F^{I,X} = &\epsilon\omega \cdot 1 + (1-a)(1-\epsilon\omega)\tilde{s}P_F^{S,X} + (1-a)(1-\epsilon\omega)\tilde{i}P_F^{I,X} + a(1-\epsilon\omega)\tilde{s}b \cdot 0 + \\ &a(1-\epsilon\omega)\tilde{s}(1-b)P_F^{S,Z} + a(1-\epsilon\omega)\tilde{i}P_F^{I,Z}, \end{aligned} \tag{S8}$$

$$\begin{aligned} P_k^{I,Z} = &\epsilon\omega \cdot 0 + \rho(1-a)(1-\epsilon\omega)\tilde{s}P_k^{S,X} + \rho(1-a)(1-\epsilon\omega)\tilde{i}P_k^{I,X} + [1-\rho(1-a)](1-\epsilon\omega)\tilde{s}b \cdot 1 + \\ &[1-\rho(1-a)](1-\epsilon\omega)\tilde{s}(1-b)P_k^{S,Z} + [1-\rho(1-a)](1-\epsilon\omega)\tilde{i}P_k^{I,Z}, \end{aligned} \tag{S9}$$

$$\begin{aligned} P_F^{I,Z} = &\epsilon\omega \cdot 1 + \rho(1-a)(1-\epsilon\omega)\tilde{s}P_F^{S,X} + \rho(1-a)(1-\epsilon\omega)\tilde{i}P_F^{I,X} + [1-\rho(1-a)](1-\epsilon\omega)\tilde{s}b \cdot 0 + \\ &[1-\rho(1-a)](1-\epsilon\omega)\tilde{s}(1-b)P_F^{S,Z} + [1-\rho(1-a)](1-\epsilon\omega)\tilde{i}P_F^{I,Z}. \end{aligned} \tag{S10}$$

Solving the above system of 8 simultaneous equations in 8 unknowns (Equations S3-S10) would lead to a closed form expression for each of $P_F^{S,X}$, $P_F^{S,Z}$, $P_k^{S,X}$, $P_k^{S,Z}$, $P_F^{I,X}$, $P_F^{I,Z}$, $P_k^{I,X}$ and $P_k^{I,Z}$. In turn this would lead to a closed-form for the p.m.f. (Equation S2), from which its expected value ($x(\tilde{i})$) could be calculated.

3 Is it possible to find a closed-form expression for $x(\tilde{i})$?

Equations S3-S10 can be expressed in matrix-vector form as

$$A\mathbf{v} = \mathbf{b}, \tag{S11}$$

where

$$A = \begin{bmatrix} B & 0 \\ 0 & B \end{bmatrix}, \tag{S12}$$

$$\mathbf{b} = \begin{bmatrix} 0 & -a(1-\epsilon\omega)\tilde{s}b & -(1-\rho)(1-\omega)\tilde{s}b & -(1-\rho(1-a))(1-\epsilon\omega)\tilde{s}b & -\omega & -\epsilon\omega & -\omega & -\epsilon\omega \end{bmatrix}^T, \tag{S13}$$

$$\mathbf{v} = \begin{bmatrix} P_k^{S,X} & P_k^{I,X} & P_k^{S,Z} & P_k^{I,Z} & P_F^{S,X} & P_F^{I,X} & P_F^{S,Z} & P_F^{I,Z} \end{bmatrix}^T, \tag{S14}$$

and where

$$B = \begin{bmatrix} (1-\omega)\tilde{s}-1 & (1-\omega)\tilde{i} & 0 & 0 \\ (1-a)(1-\epsilon\omega)\tilde{s} & (1-a)(1-\epsilon\omega)\tilde{i}-1 & a(1-\epsilon\omega)\tilde{s}(1-b) & a(1-\epsilon\omega)\tilde{i} \\ \rho(1-\omega)\tilde{s} & \rho(1-\omega)\tilde{i} & (1-\rho)(1-\omega)\tilde{s}(1-b)-1 & (1-\rho)(1-\omega)\tilde{i} \\ \rho(1-a)(1-\epsilon\omega)\tilde{s} & \rho(1-a)(1-\epsilon\omega)\tilde{i} & (1-\rho(1-a))(1-\epsilon\omega)\tilde{s}(1-b) & (1-\rho(1-a))(1-\epsilon\omega)\tilde{i}-1 \end{bmatrix}. \tag{S15}$$

If we define

$$\mathbf{b}_1 = \begin{bmatrix} 0 & -a(1-\epsilon\omega)\tilde{s}b & -(1-\rho)(1-\omega)\tilde{s}b & -(1-\rho(1-a))(1-\epsilon\omega)\tilde{s}b \end{bmatrix}^T, \tag{S16}$$

$$\mathbf{b}_2 = \begin{bmatrix} -\omega & -\epsilon\omega & -\omega & -\epsilon\omega \end{bmatrix}^T, \tag{S17}$$

and

$$\mathbf{v}_1 = \begin{bmatrix} P_k^{S,X} & P_k^{I,X} & P_k^{S,Z} & P_k^{I,Z} \end{bmatrix}^T, \tag{S18}$$

$$\mathbf{v}_2 = \begin{bmatrix} P_F^{S,X} & P_F^{I,X} & P_F^{S,Z} & P_F^{I,Z} \end{bmatrix}^T, \tag{S19}$$

then it is possible to decouple the 8 simultaneous equation system into two separate 4 equation systems, with

$$B\mathbf{v}_1 = \mathbf{b}_1, \tag{S20}$$

$$B\mathbf{v}_2 = \mathbf{b}_2. \tag{S21}$$

However it is not possible to easily find a closed-form expression for the inverse of the matrix B. This means it is impossible to write closed-form expressions for the quantities $P_F^{S,X}$, $P_F^{S,Z}$, $P_k^{S,X}$, $P_k^{S,Z}$, $P_F^{I,X}$, $P_F^{I,Z}$, $P_k^{I,X}$ and $P_k^{I,Z}$, and so in turn the p.m.f. can only be calculated numerically. There is therefore no closed-form solution for $x(\iota)$, and so the expected value of the p.m.f. would have to be calculated numerically at every time point during an epidemic (since the matrix B depends on the current state of the system via $\tilde{s}$ and $\tilde{i}$). This is certainly possible using a computer, but no further mathematical analysis appears to be possible.

References

1. Donnelly R, Cunniffe NJ, Carr JP, Gilligan CA. Pathogenic Modification of Plants Enhances Long-Distance Dispersal of Nonpersistently Transmitted Viruses to New Hosts. Ecology. 2019;100(7):e02725. doi:10.1002/ecy.2725.
