# Supplementary material for: Why aphid virus retention needs more attention: Modelling aphid behaviour and virus manipulation in non-persistent plant virus transmission
Source: PLoS Comput Biol. 2024 Oct 1;20(10):e1012479. doi: 10.1371/journal.pcbi.1012479 (PMC11469505; doi:10.1371/journal.pcbi.1012479)
Supplement: Attachment — Submitted filename: Resubmission_Cover_letter_PLOS_FINAL.pdf [file pcbi.1012479.s005.pdf]

Elin Falla
Department of Plant Sciences
University of Cambridge
Downing Street
Cambridge, CB2 3EA

UNIVERSITY OF CAMBRIDGE
Department of Plant Sciences

13th August 2024

Manuscript PCOMPBIOL-D-24-00836

Why aphid virus retention needs more attention: modelling aphid behaviour and virus manipulation in non-persistent plant virus transmission

Dear Claudio José Struchiner, M.D., Sc.D. and Virginia Pitzer,

Thank you for handling our manuscript, and for securing such useful reviews. Please find below our responses to the reviewers' comments. Note that all line numbers below refer to the version of the new manuscript showing "*Track Changes*", which allows you to see the precise alterations to the manuscript as implemented.

The only changes we made that aren't mentioned in our responses to reviewers below were (1) to remove the comma after 'i.e.' where it was present, for consistency in the manuscript and (2) to fix one reference (reference #40) that we noticed was formatted incorrectly in the Bibliography.

Thank you once again,

Elin Falla (on behalf of both authors)

Reviewer #1

The authors developed and analyzed models for a very important system: plant epidemics caused by viruses that are non-persistently transmitted (NPT) by insect vectors (primarily, aphids). Plant viruses cause numerous serious diseases of all crops, with epidemics often resulting in large economic losses. There are several classes of plant virus transmission by insects, and NPT may be the most common. There are unique properties of the NPT class, especially related to the high rate at which vectors acquire the virus via probing of plants (to determine if the plant is a suitable feeding host species or variety), and then the high rate at which vectors lose infectivity through feeding from plants. There have been models previously developed to better understand epidemics caused by plant viruses, especially compartmental (ODE) ones, and the authors do an excellent job of describing the relevant models. As stated here, these previous models do not take into account sufficient aspects of virus effects on vector behavior (mediated through the host plant) that can have a potentially large impact on epidemics (such as plant epidemic "size", invasion thresholds, or rates of increase in disease)

Of major importance is their ingenious approach to parameterizing plant attractiveness to vectors and plant acceptability once probing of the plant occurs. These are key aspects of the virus-induced vector phenotypes of "attract-and-deter" and "attract-and-retain". These behaviors have been documented in recent biological studies, and results from the modelling work here show the

Elin Falla
Department of Plant Sciences
University of Cambridge
Downing Street
Cambridge, CB2 3EA

large impact of these behaviors on plant epidemics. This is highly relevant in (ultimately) developing and testing strategies for epidemic control, although control is not part of this manuscript. Also ingenious is the derivation of a variable vector dispersal rate and the variable loss of infectivity term.

Response: We thank Reviewer 1 for their complimentary assessment of our "*ingenious*" approach to both parameterising plant attractiveness and acceptability, and to deriving variable aphid dispersal and infectivity loss rates. We are pleased to hear that the reviewer feels the results of this study are "*highly relevant in… developing and testing strategies for epidemic control*". Although the reviewer has correctly identified that control is not an explicit focus of the m/s, we note that the 'Future work and potential extensions' section of the Discussion describes extending the model to test control mechanisms for aphids and NPT viruses, by addition of resistant attractive 'trap' plants to sanitise the aphids of the virus.

Action: None (none requested)

An additional significant contribution is their derivation of the basic reproduction number, Ro, using the 'next generation' method (which I consider to be the best approach for this derivation). They explain their Ro terms in a clear and logical manner.

Response: We again thank Reviewer 1 for appreciating the "*significant contribution*" of our derivation of R0 in Appendix S2, and our "*clear and logical*" explanation of its derivation in the main text.

Action: None (none requested)

The authors appropriately discuss ways that the modeling will be extended in future work. One scenario mentioned on page 21 deals with the consideration of transitory vectors rather than colonizing vectors. I hope the authors do work on this since it is common for plant epidemics to occur due to the transmission of NPT viruses by transitory (non-colonizing) vector species, often multiple different aphid species moving through fields at the same time (where none of the aphid species colonize the plants). This might be a good place to cite Marcus & Raccah (1986; J. Appl. Stat. 13: 167-175). I know this is ancient history (!), but these authors do develop a simple discrete-time model for NTP viruses, where there is a combination of colonizing and transitory vectors. Although crude compared to the new work in the current manuscript, I do see relevance for the discussion.

Response: We thank the reviewer for acknowledging our inclusion of a transient aphid scenario as a potential future model extension. We too believe it is an important topic for modellers to consider, and indeed one of us has already begun to do so in other work published in this journal (Zaffaroni et al. (2021) *PLOS Computational Biology*. 17:e1009727). However, we were not aware of the – useful – Marcus & Raccah (1986) reference and thank the reviewer for bringing it to our attention. We have added it in as a reference to bolster our point that previous models considering transient aphids – including Zaffaroni et al. above – do not consider aphid feeding behaviour (and that it therefore should be a focus going forward).

Action: Text edit/reference added to L694-696.

Elin Falla
Department of Plant Sciences
University of Cambridge
Downing Street
Cambridge, CB2 3EA

UNIVERSITY OF CAMBRIDGE
Department of Plant Sciences

Reviewer #2

This manuscript explores whether previous approaches to model vector-borne diseases caused by non-persistent viruses (NPV) in plants can be unified in a single framework. More specifically, most ODE models (e.g., Hamelin et al 2023) account for vector preferences but ignore other NPV specifics, including the distinction between probing and feeding (Cunniffe et al 2021 is an exception). Probing may be sufficient to acquire and transmit the virus, but the vector may decide to stay longer and feed, depending on whether the plant is infected or not. In addition, virus loss is more likely on susceptible plants compared to infected plants (in which reacquisition of the virus can occur). Therefore, the rate at which vectors lose the virus and the number of plant visits per unit time (termed the "dispersal rate" in this study, even though the model is nonspatial) are dependent on S and I (the densities of susceptible and infected hosts), which is not accounted for in traditional ODE models. Donnelly et al (2017) tackled this issue in an original way, using Markov Chain theory. However, their model assumed fast vector infection dynamics relative to plant infection dynamics, which makes sense, but is less general than Cunniffe et al (2021), who additionally considered conditional vector preferences and vector population dynamics. The model presented in this study bridges the gap between Donnelly et al (2017) and Cunniffe et al (2021). The main message is that NPV specifics matter, and can be accounted for in a relatively simple ODE model. Overall, this work constitutes a useful contribution to the field of mathematical and computational plant disease epidemiology.

The manuscript is very well written, and contains many results/figures (including compelling mathematical results in appendix). My comments below intend to improve their presentation.

Response: Thank you to Reviewer 2 for recognising our work is a "*useful contribution*" to the field, and particularly for the comment appreciating the care we put into the manuscript and the "*compelling*" mathematical proofs in the Appendices.

As the reviewer has identified, we use "*dispersal rate*" in this study to denote the average number of dispersal flights per aphid per day, between pairs of plants in the same field. Although the model is non-spatial, in common with other ODE models of epidemics, it conceptualises spread through a population of <u>individual</u> distinct (plant) hosts, and so we feel this nomenclature is appropriate. This was already explained in the manuscript, e.g., in the Methods (L131-133) and Table 2, but we have added (1) a more explicit description of the precise meaning at the first point the term "*dispersal rate*" is used in the manuscript (in the Introduction), (2) specified that the dispersal rate is specifically 'between plants' in Table 2.

Action: Text edit on L60-61, minor edit to Table 2.

- In the Abstract, the sentence starting with "Additionally" sounds contradictory with the previous sentence. I would suggest replacing "Additionally" with "However" and deleting "However" from the next sentence.

Response: Thank you for this feedback – we acknowledge our initial wording was confusing. The confusion comes because we didn't clarify that the experimental evidence suggests aphids have multiple infective probes only in the case that they do not feed. Rather than replacing 'additionally' with 'however', we clarified that the experimental evidence suggests that aphids could inoculate multiple plants only in the case they do not feed on them, so now the sentence no longer sounds contradictory. We then slightly changed the wording in a couple of other places to keep the abstract at 250 words. After clarifying we believe the wording makes more sense.

Elin Falla
Department of Plant Sciences
University of Cambridge
Downing Street
Cambridge, CB2 3EA

Action: Minor changes to Abstract wording

- L15: I would suggest replacing "subsequent" with "uninfected" since reacquisition of the virus can occur on infected plants.

Response: Thank you – this comment highlighted to us that the sentences defining the NPT virus transmission process (now L13-22) was unclear. See our response to the next comment below for the changes we made to this paragraph to address this.

Action: See below

- L132-135: you state that "infective aphid feeding on a plant will always cause it to lose infectivity". This sounds like a very strong assumption when the plant is infected. Cannot the aphid acquire the virus again when feeding? I would assume the sap the aphid feeds on also contains viral particles, doesn't it? Similarly, you state "in order for virus acquisition to occur the aphid must reject (i.e., not feed on)" the plant host. I think these assumptions should be more explicitly justified, right from the Introduction (around L15 and reference [8,9]), since they are key to understand the model then.

Response: Thank you for this comment – as mentioned in response to the above comment we have added additional detail to our explanation of NPT virus transmission by aphids in the Introduction (L13-22) to make our reasons for making these assumptions clearer. Feeding on any plant, infected or not, causes ejection of saliva that washes NPT virions out of the aphid's stylet. Indeed, much experimental evidence shows NPT acquisition/retention by aphids requires probing without subsequent feeding (see references cited on L16 and L19). Inspired by this comment, and the need identified by the reviewer to make the basis of our model assumptions more explicit, we have also clarified in the Introduction and Discussion where we talk about consecutive infective probes, that this is explicitly in the case that aphid feeding does not occur (L88-94 and L550-555).

Action: Text edit/addition on L13-22. Also text edits at L88-94 and L550-555

- L153: in the Introduction, you made the point that "attract and retain" could be a way for the virus to increase its transmission provided "retain" means the growth of a local colony and the production of winged individual when the local density is high enough. But then you assume the aphid population is a constant, and is composed of winged individuals only. What is the point of considering "retain" in this model then?

Response: Thank you for this insightful comment. We carefully considered whether to include aphid population dynamics as well as the possibility of winged and wingless aphid morphs, but decided against it as it makes comparing the MIP-BAR model to the previous MIP and BAR models much more complicated, and at the cost of understandability to the reader. We believe our model usefully looks at the 'retain' phenotype even without aphid population dynamics, as the hypothesis that this phenotype causes increase in aphid numbers, and therefore increased virus transmission, is more relevant to larger landscape-scale disease spread, and therefore less relevant to our field-scale model. Additionally, our model presents novel insights into how winged aphid dispersal and feeding behaviour changes due to this 'attract-and-retain' phenotype. We show that relaxing the assumption of aphids losing infectivity after a single probe of a healthy plant increases epidemic size for all virus manipulation scenarios, but in particular for the 'retain' phenotype. This result would not change with the addition of aphid population dynamics, as it

Elin Falla
Department of Plant Sciences
University of Cambridge
Downing Street
Cambridge, CB2 3EA

shows how the behaviour of the dispersing winged aphids, and resulting infective period, responds to the ‘retain’ strategy at a local scale, independent of aphid population size.

We also already mention, in the Discussion, that the addition of aphid population dynamics to further look at the ‘retain’ strategy would be a useful addition. However, in response to this comment, we have made it clearer in this section (L707-710) that our model results corroborate previous findings that at local field-scales the ‘retain’ phenotype limits NPT virus spread. Overall, we believe that the advantages of omitting aphid population dynamics for ease of comparison of our model to previous models outweighs the advantages of its inclusion for the questions we are trying to answer in this study.

Action: Text edit on L707-710.

- L193: I would have appreciated a rederivation of Eq. (9), in a box or at least in an appendix, for the manuscript self-sufficiency, especially since the Markov Chain is alluded to in Fig. 2c. I realize that the appendix of Donnelly et al (2017) may not be that amenable to simplification though. Would it be possible to provide the reader with an intuition about the interpretation of Eq. (9)?

Response: The reviewer is correct that there is no easy way to simplify Appendix 1 of Donnelly et al. 2019 where the derivation of x(i,s) is explained. We considered including the initial derivation in an appendix, but since this would essentially replicate work from another publication, we felt this was overkill given the implicit flexibility offered by the reviewer (“*may not be that amenable to simplification though*”). However, we agree that for manuscript self-sufficiency, an explanation of the biological meaning of x(i,s) is very useful, and so we opted to add this to the main part of manuscript to give readers an easy way to interpret x(i,s) without having to refer to an appendix. To do this, we slightly rearranged the order of the section for comprehensibility.

Action: Added text and rearranged text/equations on L201-206.

- L199: Ideally, I would have liked the authors to present the most general (termed “MIP-BAR”) model first, and then show how it simplifies to previous subcases (MIP) and (BAR). The authors do a good job at showing that BAR corresponds to a slow/fast approximation of MIP-BAR, but whether MIP can be viewed as a special case of MIP-BAR is left implicit. I understand that this is not the case (due to the epsilon factor associated with v among other things), but this might be clarified.

Response: Thank you for this comment – while both initially and again in response to this comment we did consider presenting the new MIP-BAR model first, we found that it was much easier to understand where the structure of the MIP-BAR model came from when we started by explaining the two models (MIP and BAR) it was based upon. The MIP-BAR model uses the basic structure of the MIP model but with the variable rates of the BAR model. We therefore found we could more easily describe which features of each of those models we were using by referring back to them.

Additionally, as the reviewer correctly states, the MIP model is not a special case of the MIP-BAR model. In our initial submission we showed this implicitly through our results, namely Figures 5 and 6. There is no systematic way to match the MIP and MIP-BAR models due to the variable dispersal and infectivity loss rates in MIP-BAR model, which are dependent on the stage of the

Elin Falla
Department of Plant Sciences
University of Cambridge
Downing Street
Cambridge, CB2 3EA

epidemic. The MIP model has constant dispersal and infectivity loss rates for all stages of the epidemic, so over a time course the two models can never be exactly matched in a systematic way. However, the reviewer makes a good point that we don't explicitly say that the MIP model isn't a special case of MIP-BAR, so we've added a more detailed explanation of their differences in the results (L346-354), and explicitly stated this in the discussion (L498-500).

Action: Added text - L346-354 and L498-500

- L592-595: I wonder whether this observation could be an artifact due to the fact that the travel time between plants is assumed to be zero. I would appreciate a discussion around this assumption (i.e., the "shorter average infective period" does not account for travel times, so it should not be interpreted in a straightforward way).

Response: This is an astute comment. It is true that if the flight length between plants is considered, the infective period would be longer than our model suggests when aphid dispersal is increased. However, given that aphids are generally only in appetitive flight between plants in the same field for at most up to 5 minutes at a time (Parry et al. 2013 – now reference #43 in m/s), whereas aphids feed for several hours (Powell et al. 2006), adding in flight time would only have a very marginal effect on infective period length. We therefore feel justified in omitting this from our model and analysis. However, we agree that a discussion around this is useful, so we have added text to the Discussion.

Action: Added text and reference (43) - L626-632

A more general comment has to do with the fact that many results are presented, some being more expected/intuitive than others. It is difficult to quickly grasp the most salient results from the manuscript in its current form. Please consider a way to improve this.

Response: Thank you for this comment. We do present a lot of results, but our defence is that this is merely a reflection of the depth of our ambition in this paper!

In response to this comment, we have added to the Conclusions section (L717-743), drawing out the key results more explicitly and referring back to the Figures that they relate to.

We also note throughout the manuscript we have already used various techniques to distil the information into an easily consumable format. These include

- Naming the models by their features (Table 1 in manuscript) i.e. the MIP model, BAR model and our MIP-BAR model that combines features of both previous models.
- Organising our results into answers to three 'key questions' (Qs) which we introduce at the end of the Introduction. The Results and Discussion sections are split by these same key questions, with the sections titled as the answer to these questions. The 3 key questions split the results into comparing MIP vs MIP-BAR (Figures 5 + 6), BAR vs MIP-BAR (Figure 7), then insights from the MIP-BAR model (Figure 8).
- Moving our more mathematical, less immediately digestible results into Appendices (namely Appendices S1 and S3).

In reflecting on this comment, we did consider other ways to structure our work. For example, we could present our MIP-BAR model first (Figure 8 first), then the comparison to previous models. However, we feel the comparison to previous models, particularly to MIP (Figures 5 + 6) gives

Elin Falla
Department of Plant Sciences
University of Cambridge
Downing Street
Cambridge, CB2 3EA

important context that makes the later MIP-BAR model results more understandable. We considered putting Figure 4 panels b and c – which explain why the MIP-BAR and BAR model trajectories can be matched so closely – in an Appendix, as we did with the maths explaining why their equilibria always match when $\rho = 1$ (Appendix S1). However, we ultimately decided that this would be reducing too much of the explanation of the similarities between these models to Appendices, resulting in a less clear understanding.

Therefore, we have decided to leave the overall structure as it is for now, but are happy to revisit this point – if necessary – following further input from the Editor.

Action: Edited/added to text – L717-743
